# B-Site Mixing Effects in Hybrid Perovskites: Phase Transitions and Dielectric Response of MAPb_1–*x*_Sn_*x*_Br_3_

**DOI:** 10.1021/acs.chemmater.4c03381

**Published:** 2025-01-29

**Authors:** Gabrielius Rimkus, Sergejus Balčiu̅nas, Hanna R. Petrosova, Olesia I. Kucheriv, Rokas Lemežis, Vytautas Klimavičius, Vidmantas Kalendra, Ju̅ras Banys, Il’ya A. Gural’skiy, Mantas Šimėnas

**Affiliations:** † Faculty of Physics, Vilnius University, Sauletekio 3, LT-10257 Vilnius, Lithuania; ‡ Department of Chemistry, Taras Shevchenko National University of Kyiv, Kyiv 01601, Ukraine; § Institute of Chemical Physics, Vilnius University, Sauletekio 3, LT-10257 Vilnius, Lithuania

## Abstract

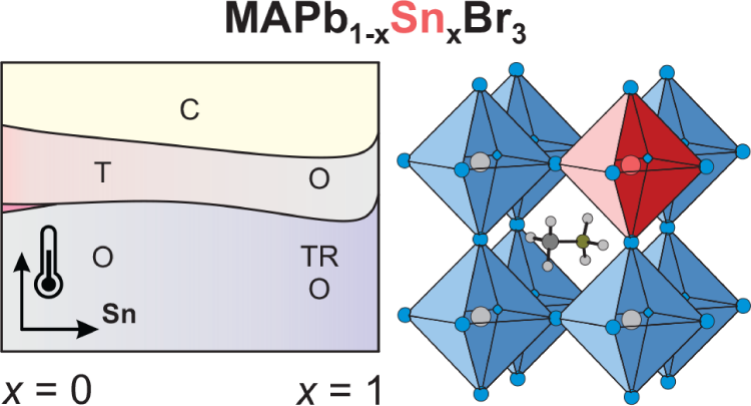

Ion-mixing is a highly effective strategy for tuning the performance and stability of photovoltaic devices based on hybrid perovskites. Despite many works concentrating on the A- and X-site mixing effects, a comprehensive study on the effects of B-site mixing on the structural and dynamic properties of MA-based perovskites is still absent. In this work, we investigate the structural and dynamic properties of mixed lead–tin halide perovskites MAPb_1–*x*_Sn_*x*_Br_3_ using a multitechnique experimental approach including differential scanning calorimetry, dielectric spectroscopy, and nuclear quadrupole resonance experiments. We map the phase diagram of this system, which reveals that B-site mixing slightly stabilizes the cubic phase and affects the MA cation dynamics and ordering, although the observed effects are less prominent compared with A- and X-site mixing. Our results provide insights into the complex interplay of structural and dynamic properties in mixed-metal perovskites contributing to their potential optimization for photovoltaic applications.

## Introduction

Hybrid metal halide perovskites have attracted exceptional attention as highly efficient and solution-processable materials for photovoltaic applications.^1,2^ In the past decade, the power conversion efficiency of solar cells based on methylammonium (MA, CH_3_NH_3_^+^) lead iodide MAPbI_3_ and related compounds has demonstrated an exceptional increase and currently exceeds 25%.^3−8^ Despite their remarkable performance, the applicability of lead-based photovoltaic devices faces a lead toxicity challenge. Thus, perovskite compositions with more environmentally friendly alternatives are also widely investigated including tin-based compounds such as MASnX_3_ (X = I, Br).^9−16^ In addition to lower toxicity, tin-based perovskites also have the potential for higher charge carrier mobility.^12,17^ However, currently, the mobility in these compounds still suffers from the oxidation of Sn^2+^ to Sn^4+^, which leads to self-doping.^10,17^

In general, the highest performance and stability of hybrid perovskites are obtained for compositions with mixed molecular cations (A-site) and halide anions (X-site).^18,19^ In addition to the device performance, the ion-mixing in these compounds also significantly affects their structural and dynamic properties.^20^ As demonstrated in previous works by us and other groups, mixing at the A- and X-site typically results in lattice symmetrization and the formation of a glassy dipolar phase caused by a suppression of the structural phase transitions occurring in these materials.^20−28^ In addition, mixing in hybrid perovskites can also be used as a strategy to significantly tune the dielectric properties relevant for photovoltaic applications.^22−24,26,29,30^

Despite many works concentrating on the A- and X-site mixing effects, a comprehensive study on the B-site mixing on the structural and dynamic properties of hybrid perovskites is still absent.^20^ In this work, we use a suite of experimental techniques to investigate a family of mixed MAPb_1–*x*_Sn_*x*_Br_3_ perovskites. Our approach allows us to map the temperature–concentration phase diagram of these compounds, which reveals a relatively weak effect on the structural phase transitions even for the highest mixing levels. We also observe signatures of the glassy phase of electric dipoles, suggesting a disruption of the molecular cation dynamics and ordering upon mixing. In general, our findings indicate that mixing at the B-site has weaker effects compared to A- and X-site mixing.

## Experimental Details

### Sample Synthesis

The quantities of precursors used for MAPb_1–*x*_Sn_*x*_Br_3_ synthesis are given in Table S1. All studied samples were obtained by the following procedure: tin(II) chloride was dissolved in H_2_O with a minor quantity of HCl (38%) to prevent hydrolysis. Next, ammonia solution was added under continuous stirring and cooling in an ice bath. As a result, a white solid precipitate of Sn(OH)_2_ was formed. The resulting precipitate was carefully separated by filtration and thoroughly washed with water. The resulting tin hydroxide was dissolved in a cold mixture consisting of hydrobromic acid (48%) and H_3_PO_2_. Lead bromide was dissolved in hydrobromic acid (48%) and added to the previous solution. Methylammonium bromide was added to a solution of metal bromides under continuous stirring. A precipitate of MAPb_1–*x*_Sn_*x*_Br_3_ was immediately formed and was quickly filtered off. The precipitate was then carefully washed with an acidified methanol solution (a mixture of methanol and hydrobromic acid in a 10:1 ratio). Finally, the precipitate of MAPb_1–*x*_Sn_*x*_Br_3_ was dried in a vacuum for 2 h. The losses during the synthesis resulted in a yield of 60–70% depending on the ratio of the metals.

### PXRD

Powder X-ray diffraction (PXRD) patterns were obtained on a Shimadzu XRD-6000 diffractometer using Cu-Kα radiation (5–50° range, 0.05° step) at room temperature.

### DSC

Differential scanning calorimetry (DSC) measurements were performed on a Linkam DSC600 instrument. Before starting the heating/cooling experiment, the stage chamber was purged with dry nitrogen for 5 min. The measurements were carried out in the temperature range from 88 to 298 K with a heating/cooling rate of 10 K/min.

### SEM and EDX Measurements

Energy-dispersive X-ray spectroscopy (EDX) was used to determine the elemental composition of the materials. The EDX analysis of the MAPb_1–*x*_Sn_*x*_Br_3_ samples was carried out using an energy-dispersive X-ray spectroscopy analyzer (EDAX Octane Elite) attached to a Verios G4 UC scanning electron microscope (SEM).

### Dielectric Spectroscopy

The dielectric spectroscopy experiments were performed in the 100 Hz to 1 MHz frequency range using an HP4284A LCR meter. A flat capacitor model was used to calculate the complex dielectric permittivity from the measured capacitance and loss tangent of the pressed pellet samples. Temperature-dependent dielectric spectra were measured on cooling at a rate of less than 1 K/min. Silver paste was used as sample electrodes.

### NQR

^81^Br nuclear quadrupole resonance (NQR) measurements were carried out on a 400 MHz Bruker AVANCE III HD NMR spectrometer. The Bruker low-temperature wide-line single-channel probe coupled with a JANIS helium open-cycle cryostat placed outside the NMR magnet was used. ^81^Br NQR measurements were carried out at 295, 190, and 100 K using piecewise acquisition in steps of 150 kHz using a Hahn-echo pulse sequence (π/2 - delay - π - acquisition). The total covered spectral range was 57.43–75.38 MHz. The excitation pulse was set to 1.675 μs (150 kHz excitation bandwidth), the delay between excitation and refocusing pulses was 32.5 μs, the repetition delay was set to 0.1 s, and 2048 scans were accumulated for each subspectrum. The temperature evolution of the signal at 58.8 MHz was measured using one subspectrum in the range of 180–270 K in steps of 10 K, with 2048–8192 scans accumulated.

## Results and Discussion

**Figure 1:**
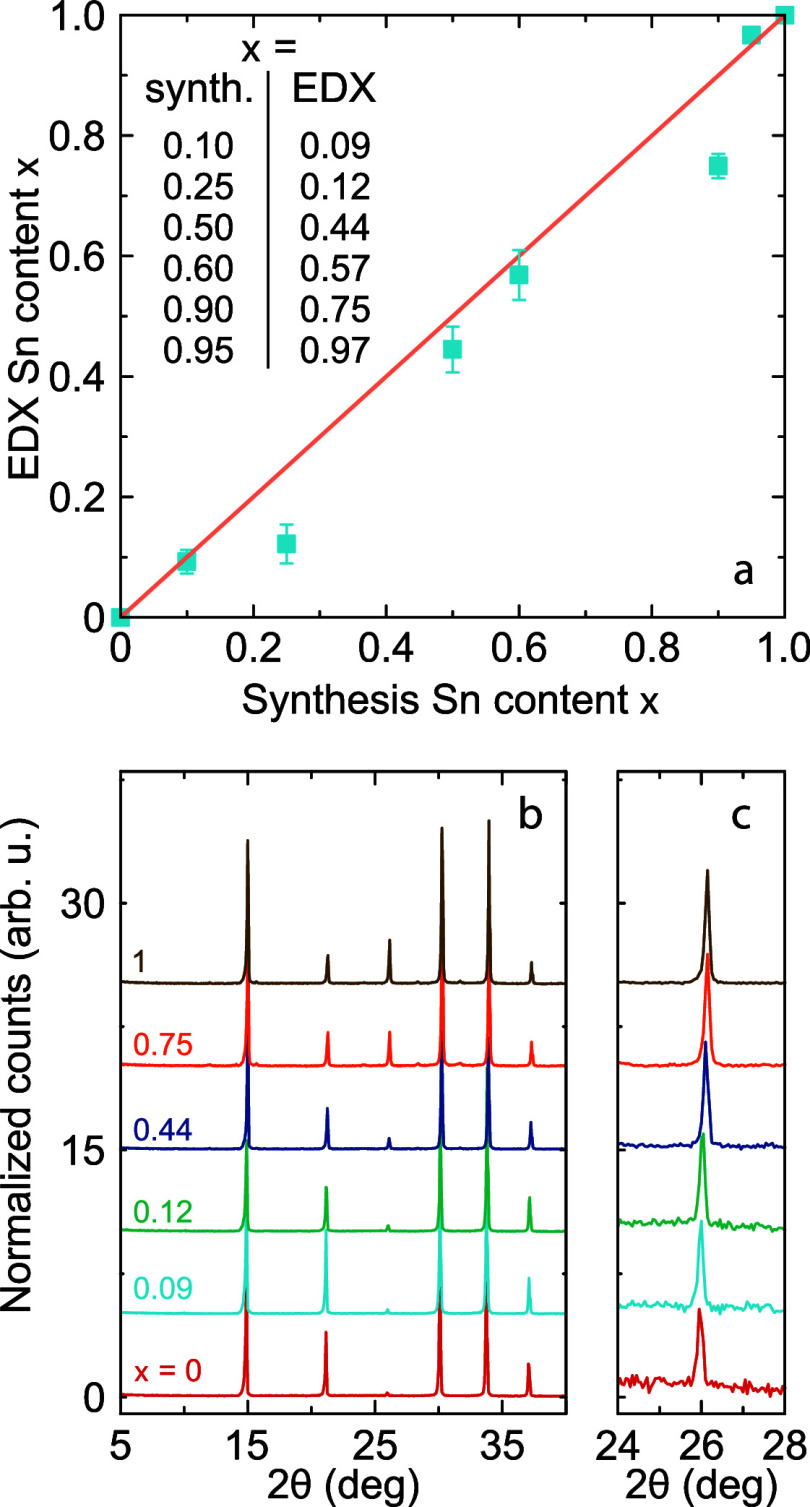
(a) A comparison of the Sn content in the synthesized samples as obtained from the EDX measurements and the ratio of the precursors used in the synthesis. The error bars were calculated by performing the EDX experiment on three different areas for each sample. (b) Room-temperature PXRD patterns of MAPb_1–*x*_Sn_*x*_Br_3_ showing the cubic *Pm*3̅*m* symmetry. (c) Upon the introduction of tin, the PXRD peaks shift gradually to higher Bragg angles.

Our study was performed on the mixed MAPb_1–*x*_Sn_*x*_Br_3_ perovskites with the nominal tin concentration of *x* = 0, 0.1, 0.25, 0.5, 0.6, 0.9, 0.95, and 1. First, we performed EDX and PXRD experiments to characterize the Pb:Sn ratio and phase mixing. Figure 1a shows the results of the EDX measurements performed on different crystallites of the synthesized samples (see Figure S1 for the SEM images, Supporting Information). The obtained results generally follow a linear trend, demonstrating a clear correlation between the Sn content in precursors and the Sn content as determined by the EDX method. The majority of points lie below the diagonal, indicating a preference for Pb incorporation, though this observation may also result from the limited precision of the EDX experiment. In the rest of this study, we will denote the tin fractions as determined from the EDX experiments: *x* = 0, 0.09(2), 0.12(3), 0.44(4), 0.57(4), 0.75(2), 0.97(1), and 1.

We also performed room-temperature PXRD measurements on the majority of the studied compounds (Figure 1b), showing the presence of a single cubic phase for all compositions in agreement with the previous study^31^ and the expected *Pm*3̅*m* symmetry of MAPbBr_3_ and MASnBr_3_.^20,32,33^ Upon the introduction of tin, the PXRD peaks gradually shift to slightly higher Bragg angles, where the most notable difference can be seen for the peak at 2θ = 25.8° (corresponding to the (111) plane) (see Figure 1c). This indicates a slightly smaller unit cell volume of MASnBr_3_ compared to MAPbBr_3_. To study the phase purity of the mixed compositions, we compared the PXRD patterns of the *x* = 0.44 compound and the 1:1 physical mixture of pure MASnBr_3_ and MAPbBr_3_ perovskites. Clearly distinct PXRD patterns were observed (see Figure S2 for details), indicating a homogeneous mixing for the studied compositions.

**Figure 2:**
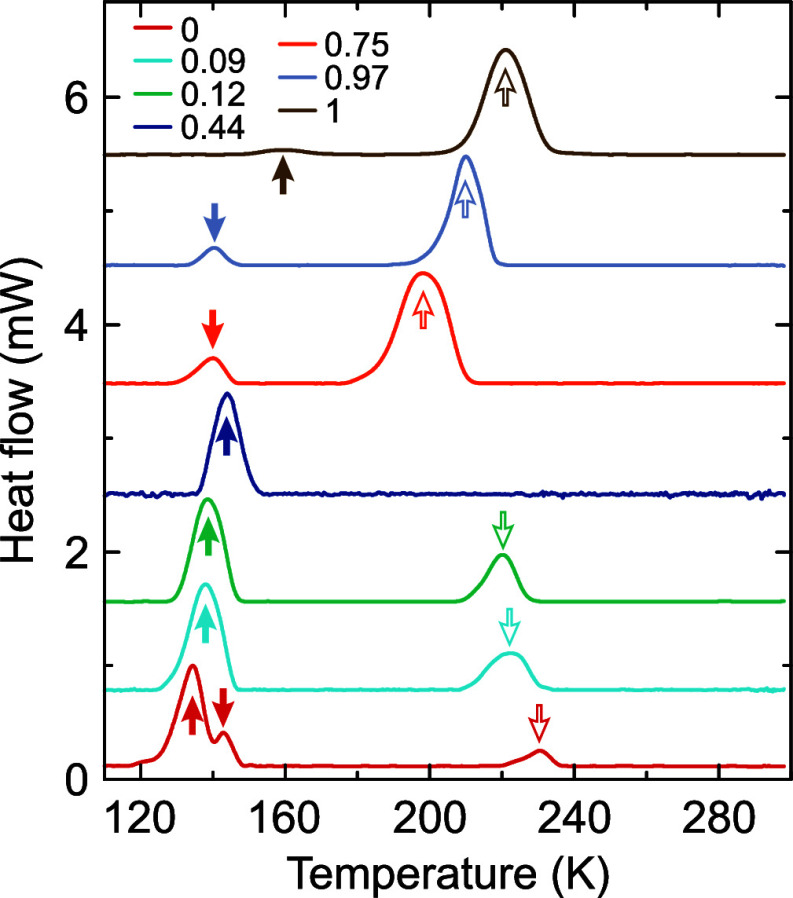
DSC traces of MAPb_1–*x*_Sn_*x*_Br_3_ samples obtained on cooling. The empty and filled arrows indicate anomalies associated with the high- and low-temperature structural phase transitions, respectively.

To investigate how the structural phase transitions are affected by the B-site mixing, we first performed DSC experiments (see Figure 2). For nonmixed MAPbBr_3_ compound, three well-documented anomalies associated with the cubic (*Pm*3̅*m*)–tetragonal (*I*4/*mcm*)–tetragonal (*P*4/*mmm*)–orthorhombic (*Pnma*) structural phase transitions are observed.^20^ Upon the introduction of tin, the temperature of the cubic–tetragonal phase transition is decreased by about 10 K (*x* = 0.12), while for the highest studied mixing level (*x* = 0.44), this transition is no longer visible in the DSC data.

The DSC data also show that the transition between the tetragonal phases is no longer resolved and thus likely suppressed upon the introduction of tin as both transitions seem to merge into a single anomaly (Figure 2). Interestingly, as the tin concentration is increased, the temperature of the transition to the orthorhombic phase slightly increases. This observation is in sharp contrast to the A-site mixing in the MAPbBr_3_-based perovskite families such as MA/DMAPbBr_3_ and MA/FAPbBr_3_, where the transition temperatures decrease drastically with the introduction of guest cations.^23,24,34^

In MASnBr_3_, the perovskite exhibits two phase transitions at about 220 and 160 K (Figure 2). The temperature of the first phase transition is in agreement with the reported calorimetric and NQR studies, while the transition point of the second transformation is slightly lower (160 K vs 180 K).^35−37^ Note that in a recent study, López et al.^38^ observed that in mechanosynthesized MASnBr_3_, the low-temperature phase transition occurs at 147 K, which may indicate that the temperature of this phase transition is susceptible to sample preparation or the size of the crystallites. The phase transitions in the Sn-rich mixed compounds (*x* = 0.97 and 0.75) occur at about 20 K lower temperatures compared to nonmixed MASnBr_3_, indicating a relatively weak effect of B-site mixing on the structural phase transitions.

Based on the synchrotron X-ray powder diffraction study by Swainson et al.,^32^ we assign these transitions to the cubic (*Pm*3̅*m*)–orthorhombic (*Pmc*2_1_)–triclinic (*P*1) symmetry lowering. Note that a more recent synchrotron study by López et al. of mechanosynthesized MASnBr_3_^38^ reported a contradictory observation, as the low-temperature phase was found to remain orthorhombic *Pmc*2_1_.

**Figure 3:**
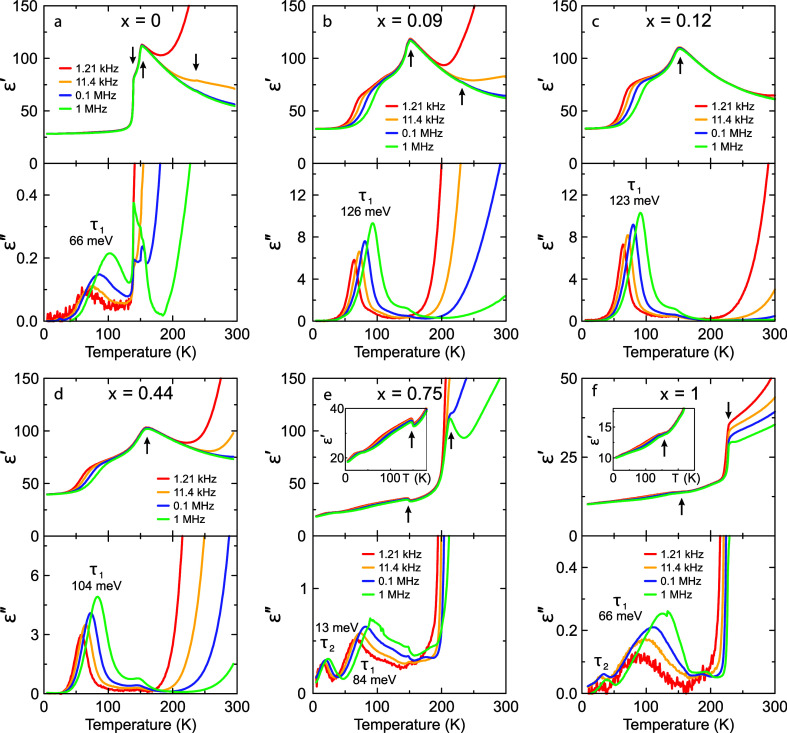
(a–f) Temperature dependence of the complex dielectric permittivity of MAPb_1–*x*_Sn_*x*_Br_3_ pellet samples presented at selected frequencies. The arrows indicate phase transition anomalies. Different relaxation processes (τ_1_ and τ_2_) are indicated, together with the determined activation energies. The insets in (e, f) emphasize the low-temperature behavior of ε′.

As demonstrated in our previous works on the A-site mixing in hybrid perovskites,^23,24,26,28^ the employment of a single experimental technique could lead to inaccurate mapping of the phase diagrams, especially in the regions of highly mixed compositions. Thus, to complement our DSC data, we also performed dielectric spectroscopy experiments in a broad temperature and frequency range. The temperature dependence of the real ε′ and imaginary ε″ (dielectric loss) parts of complex dielectric permittivity ε* = ε′ – *i*ε″ for *x* = 0, 0.09(2), 0.12(3), 0.44(4), 0.75(2), and 1 compositions is presented in Figure 3. The dielectric permittivity of the nonmixed MAPbBr_3_ perovskite exhibits a well-known sharp decrease at the tetragonal–orthorhombic phase transition point (Figure 3a) caused by the long-range cooperative ordering of the MA electric dipoles.^20,22,29,39−41^ The phase transition between the tetragonal phases results in a maximum of ε′, while the cubic–tetragonal transition is best visible in the first derivative of the dielectric permittivity data (see Figure S3).

Upon the introduction of tin, the low-temperature phase transitions merge into a single tetragonal–orthorhombic phase transition (Figure 3b–d), in agreement with the DSC data (Figure 2). For the *x* = 0.09, 0.12, and 0.44 compositions, the cubic–tetragonal phase transition is visible in the first derivative of the dielectric permittivity data (Figure S3). Note that for the *x* = 0.44 sample, the DSC anomaly associated with this phase transition was not resolved (see Figure 2).

**Figure 4:**
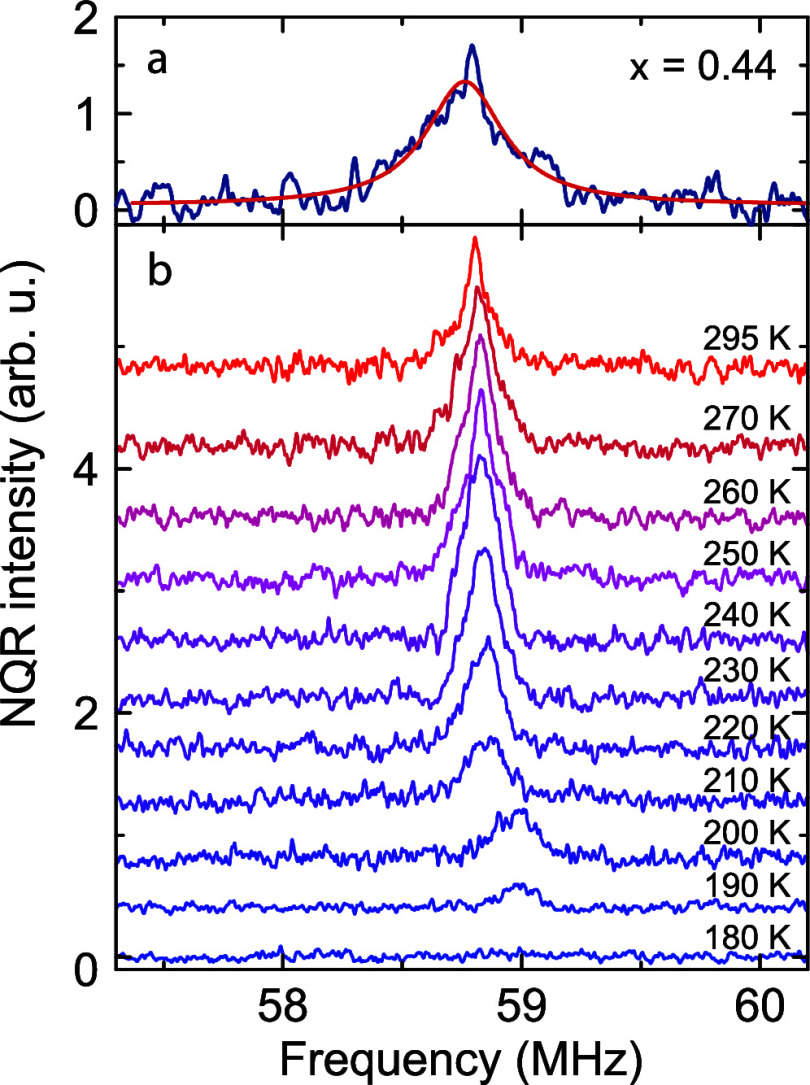
(a) ^81^Br NQR spectrum of the *x* = 0.44 sample obtained at room temperature. The red solid curve is the Lorentzian fit. (b) Temperature dependence of the ^81^Br NQR subspectrum of the *x* = 0.44 sample measured at 58.8 MHz.

To clarify this discrepancy, we also performed temperature-dependent ^81^Br NQR measurements of the *x* = 0.44 sample. At room temperature, we observed a Lorentzian NQR spectrum of ^81^Br associated with the Pb local environment (Figure 4a).^42^ Note that due to the limited bandwidth of the excitation pulses, this signal was obtained using piecewise acquisition. To clarify the presence of the phase transition, we performed temperature-dependent ^81^Br NQR experiments on a single subspectrum of the NQR line as shown in Figure 4b. It can be seen that the intensity of the signal sharply drops below 220 K and totally vanishes at 180 K. A similar behavior was also reported for the phase transition in the nonmixed MAPbBr_3_ perovskite,^42^ conforming the presence of the cubic–tetragonal transition in the *x* = 0.44 sample.

For the nonmixed MASnBr_3_ compound, the dielectric response shows both structural phase transitions (Figure 3e,f). Upon the introduction of lead (*x* = 0.75), the phase transitions slightly shift to lower temperatures, in agreement with the DSC data. Note that the dielectric permittivity value for the Sn-rich compositions (especially *x* = 1, Figure 3f) seems to be lower at low temperatures compared to the lead-based compositions, indicating a lower polarizability of the inorganic lattice.^43^

**Figure 5:**
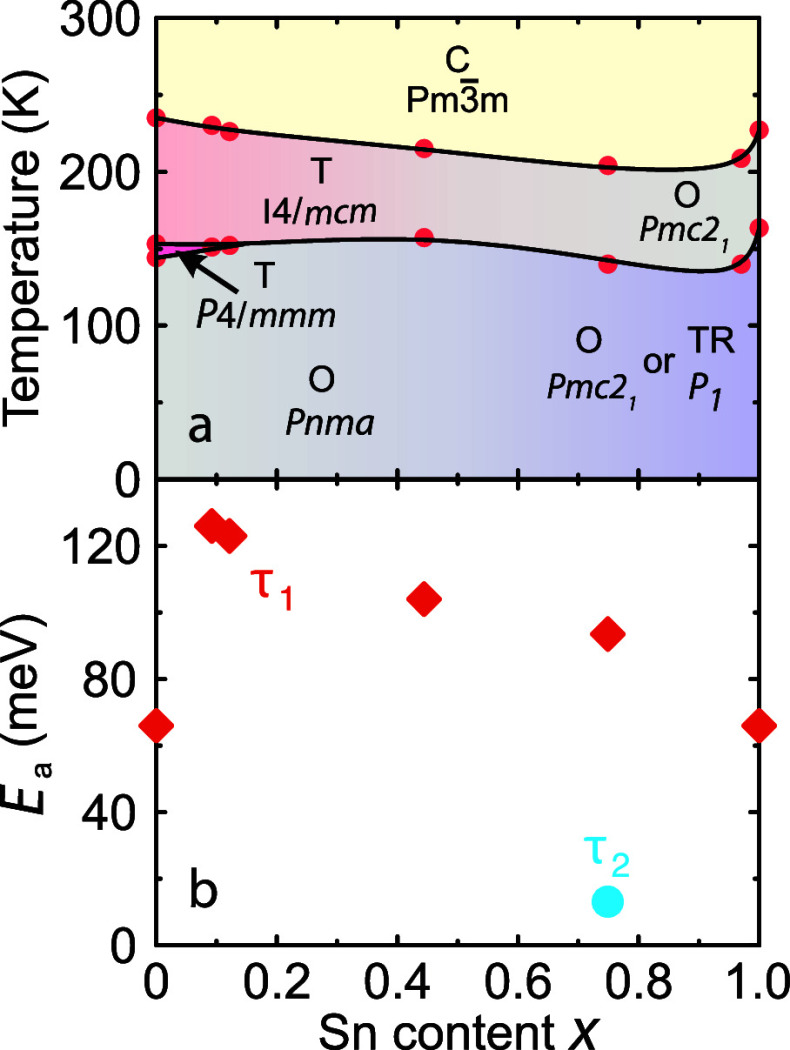
(a) Temperature–composition phase diagram of mixed MAPb_1–*x*_Sn_*x*_Br_3_ perovskites. Dots indicate structural phase transition points as determined from the DSC, NQR, and dielectric spectroscopy data. Solid curves indicate phase boundaries. Abbreviations: C = cubic, T = tetragonal, O = orthorhombic, TR = triclinic. (b) Sn concentration dependence of the activation energy of the MA cation dynamics (τ_1_) and the low-temperature process (τ_2_). The error bars are smaller than the data points.

We summarize the obtained results of the structural phase transitions in the temperature–composition phase diagram of MAPb_1–*x*_Sn_*x*_Br_3_ presented in Figure 5a. The phase diagram shows that the B-site mixing slightly increases the stability of the cubic phase, but the effect is substantially weaker than observed for the A-site mixing.^20^ The change in the transition temperature is even weaker for the low-temperature phase transition. These observations suggest a strong structural compatibility between the two perovskite systems attributed to their similar structural properties including lattice constant, symmetry, and MA cation disorder. In contrast, the structures of the end members with different A-site cations can differ significantly,^20^ leading to reduced mutual compatibility and thus significant distortion of the long-range ordering.

In addition to the phase transition anomalies, the dielectric response of MAPb_1–*x*_Sn_*x*_Br_3_ perovskites can also be used to study dynamic processes of electric dipoles. The nonmixed MAPbBr_3_ compound shows a weak dielectric relaxation in the orthorhombic phase (we denote this process as τ_1_), as is evident from the dielectric loss data (Figure 3a). This process becomes significantly stronger in the mixed compounds (Figure 3b–d), while its intensity decreases again for the Sn-rich compositions (Figure 3e,f). We assign this process to the incomplete ordering of MA cations, which enables partial reorientation dynamics. In the nonmixed MAPbBr_3_ and MASnBr_3_ compounds, such a disorder is likely caused by local lattice defects, and thus, the number of dynamically active MA cations is relatively small. In contrast, metal-mixing results in different local environments for the MA cations, substantially perturbing their long-range order and resulting in a glassy phase.^44^ Note that such a glassy behavior was also observed for the A-site^20−24,26,28^ and X-site^25^ mixing, demonstrating a universal behavior of MA cations upon mixing in hybrid perovskites.

A quantitative analysis of the dielectric response in the frequency domain allows us to obtain the activation energy *E*_a_ of the observed dipolar process τ_1_ (see Supporting Information, Figures S4 and S5). The determined activation energies for different mixed compositions are presented in Figure 5b. A sharp increase in *E*_a_ from 66(3) to 126(3) meV can be seen upon the introduction of a small amount of tin (*x* = 0.09), which is likely caused by transitioning from native to mixing-induced defects in the inorganic framework. Note that the sudden increase in *E*_a_ associated with the τ_1_ process correlates with the complete suppression of the intermediate tetragonal phase (see Figure 5a). With a further increase of *x*, the activation energy gradually decreases in the whole mixing range. Surprisingly, the *E*_a_ value for the nonmixed MAPbBr_3_ and MASnBr_3_ perovskites is practically the same, indicating very similar reorientation barriers of partially disordered MA cations in both compounds. Note that a significant increase of activation energies was also observed for mixing at the A-site in hybrid perovskites,^20,23,24,26,28^ showing that this is a universal mixing effect.

In addition to the dominant τ_1_ process, the Sn-rich compositions (*x* = 0.75 and 1) also exhibit a much weaker relaxation below 50 K, which we denote as τ_2_ (Figure 3e,f). This process is likely related to a small anomaly in the heat capacity data of MASnBr_3_ observed by Onoda-Yamamuro et al., which was tentatively assigned to a structural phase transition of a displacive type.^36^ The determination of the activation energy of this process proved to be complicated due to the overlap with another unresolved relaxation (Figure S6). However, for the *x* = 0.75 composition, we managed to extract the *E*_a_ value of about 13 meV (Figure S6), which is much smaller than that obtained for the dominant τ_1_ process (Figure 5b). This indicates that if present, the phase transition in this low-temperature region involves subtle changes in the crystal structure. The τ_2_ process may also be associated with the glassy disorder of the organic sublattice, which is not frozen at low temperatures.

## Summary and Conclusions

In this study, we investigated the structural and dynamic properties of mixed-metal MAPb_1–*x*_Sn_*x*_Br_3_ hybrid perovskites to explore the effects of B-site mixing on structural phase transitions and dipolar dynamics.

The DSC, dielectric spectroscopy, and ^81^Br NQR experiments allowed us to map the temperature–concentration phase diagram for these compounds. Our findings show that the introduction of tin into lead-based perovskites leads to a relatively weak effect on the structural phase transitions. This is in contrast to A- and X-site mixing, where a strong or even full suppression of the phase transitions can be observed.

The dielectric measurements also allowed us to study the effect of B-site mixing on MA cation dynamics. Similarly to the A-site mixing, we observed that metal-mixing significantly increases the number of dynamically active MA cations in the low-temperature phase, demonstrating a substantial disruption of the long-range ordering of the organic sublattice and formation of a glassy phase. The activation energy of the observed process also increased in the mixed compounds, indicating that mixing raises the reorientation barrier of the MA cations. For tin-rich compositions, an additional relaxation process was observed below 50 K, suggesting subtle changes in the crystal structure that may be related to a weak structural phase transition.

In conclusion, our results demonstrate that metal-mixing has a comparatively weaker impact on the structural phase transitions than A- and X-site mixing but still significantly affects the dipolar dynamics of the system. Our findings highlight the importance of understanding both structural and dynamic aspects in mixed perovskites, which influence the photovoltaic properties of these compounds.

## Supporting Information Available

Additional sample synthesis, SEM, and dielectric spectroscopy data (PDF)
