# Supplementary material for: B-Site Mixing Effects in Hybrid Perovskites: Phase Transitions and Dielectric Response of MAPb1–xSnxBr3
Source: Chem Mater. 2025 Jan 29;37(3):1314–20. doi: 10.1021/acs.chemmater.4c03381 (PMC11824424; doi:10.1021/acs.chemmater.4c03381)
Supplement: Supplementary file 1 — cm4c03381_si_001.pdf [file cm4c03381_si_001.pdf]

Supporting Information
B-site Mixing Effects in Hybrid Perovskites: Phase Transitions and Dielectric Response of $MAPb_{1-x}Sn_xBr_3$

Gabrielius Rimkus,[†] Sergejus Balčiūnas,[†] Hanna R. Petrosova,[‡] Olesia I. Kucheriv,[‡] Rokas Lemežis,[¶] Vytautas Klimavičius,[¶] Vidmantas Kalendra,[†] Jūras Banys,[†] Il'ya A. Gural'skiy,[‡] and Mantas Šimėnas[*,†]

†*Faculty of Physics, Vilnius University, Sauletekio 3, LT-10257 Vilnius, Lithuania*

‡*Department of Chemistry, Taras Shevchenko National University of Kyiv, Kyiv 01601, Ukraine*

¶*Institute of Chemical Physics, Vilnius University, Sauletekio 3, LT-10257 Vilnius, Lithuania*

E-mail: mantas.simenas@ff.vu.lt

Additional sample synthesis data

Table S1: The quantities of precursors used for the synthesis of $MAPb_{1\text{-}x}Sn_xBr_3$

x	$PbBr_2$ (mg)	$SnCl_2$ (mg)	H_2O (mL)	HCl (mL)	$NH_3 \cdot H_2O$ (mL)	HBr (mL)	H_3PO_2 (mL)	MABr (mg)
1	-	380	3.2	0.32	1.6	2.5	0.8	
0.95	36.7	361	3.04	0.304	1.52	2.375	0.76	
0.9	73.4	342	2.88	0.288	1.44	2.25	0.72	
0.75	183.5	285	2.4	0.24	1.2	1.88	0.6	
0.6	293.6	228	1.92	0.192	0.96	1.5	0.48	224
0.5	367	190	1.6	0.16	0.8	1.25	0.4	
0.25	550.5	95	0.8	0.08	0.4	0.625	0.2	
0.1	660.6	38	0.32	0.032	0.16	0.25	0.08	
0	734	-	-	-	-	0.8	-	

Additional SEM data

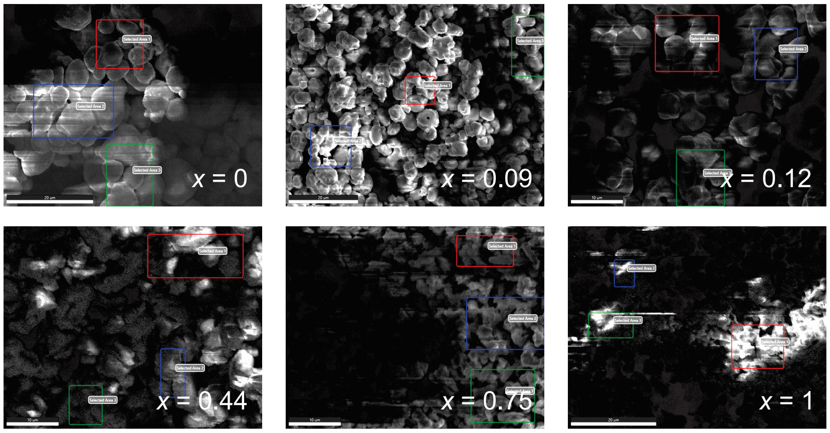

Figure S1: SEM images of $MAPb_{1\text{-}x}Sn_xBr_3$ samples. Rectangles indicate sample areas used for the EDX measurements. The indicated Sn content was determined from the EDX experiments. Note that the crystallites for the Sn-rich compositions are slightly smaller.

Additional PXRD data

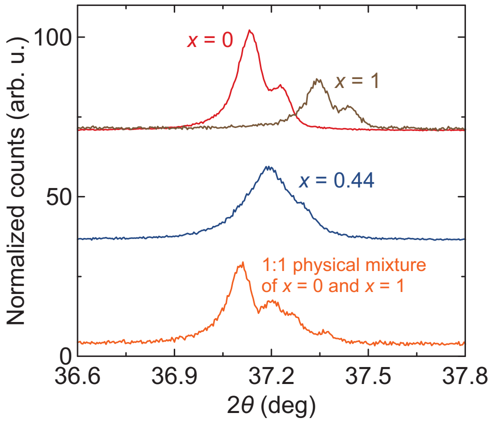

Figure S2: Room-temperature PXRD patterns centered around the $2\theta \sim 37°$ peak for the $x = 0$, 0.44 and 1 compositions. The PXRD pattern of the 1:1 physical mixture of the pure $x = 0$ and $x = 1$ perovskites is presented for comparison showing absence of the phase separation in the mixed compounds.

Additional dielectric spectroscopy data

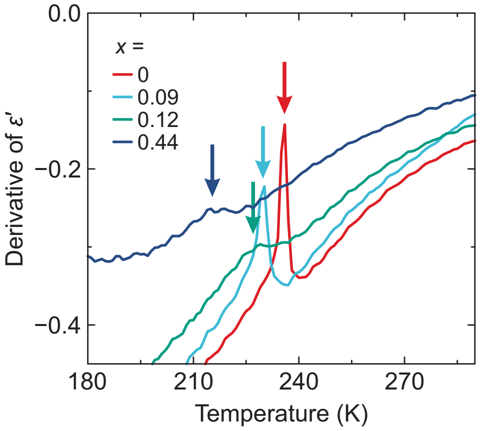

Figure S3: Temperature dependence of the first derivative of the ε' data for $x = 0$, 0.09, 0.12 and 0.44 samples obtained at 1 MHz probing frequency. The arrows indicates the cubic-tetragonal phase transition anomaly.

The frequency dependence of ε^* of $MAPb_{1\text{-}x}Sn_xBr_3$ perovskite compounds is presented in Figure S4 for selected temperatures. The observed relaxation was approximated using the Cole-Cole equation:[1]

$$\varepsilon^*(\omega) = \varepsilon(\infty) + \frac{\Delta\varepsilon}{1 + (i\omega\tau)^{1-\alpha}}. \tag{S1}$$

Here, $\varepsilon(\infty)$ is the dielectric permittivity in the high-frequency limit, $\Delta\varepsilon$ denotes the dielectric strength of the process, τ is the mean relaxation time, and $\omega = 2\pi\nu$ is the angular measurement frequency. The relaxation width is described by the parameter $0 \leq \alpha < 1$. For $\alpha = 0$, the Cole-Cole process reduces to the Debye relaxation, which describes non-interacting electric dipoles.

The best fits of Eq. S1 to the frequency domain data are also presented in Figure S4. Note that in the low-frequency region ($<$ 1 kHz), a process related to conductivity effects and charge separation can be observed, which is ignored in this study, as it is well-separated from the main relaxation. In the high-frequency region ($>$ 100 kHz), we can also observe some discrepancies between our model and the experimental data, especially for the $x = 0.09$ and 0.12 compositions. This may indicate presence of another high-frequency process or that the employed Cole-Cole empirical model is not sufficient to fully capture the unknown distribution of the relaxation times. Other models such as Havriliak-Negami[1] would provide a better fit. However, we note that it is very easy to overfit the frequency response, especially when the tails of the dielectric spectrum are not fully resolved. Thus, to avoid overfitting, we chose the simple Cole-Cole model, which is sufficient to obtain the mean dipolar relaxation time of our system.

Fitting of the data allowed us to determine the temperature dependences of the mean relaxation time τ of the observed relaxation process, as presented in Figure S5. For all studied compounds, the relaxation times follow the Arrhenius law: $\tau = \tau_0 \exp(E_a/kT)$, where E_a and τ_0 denote the activation energy and attempt time, respectively, and k is the Boltzmann constant. The determined activation energies are also listed in Figure S5.

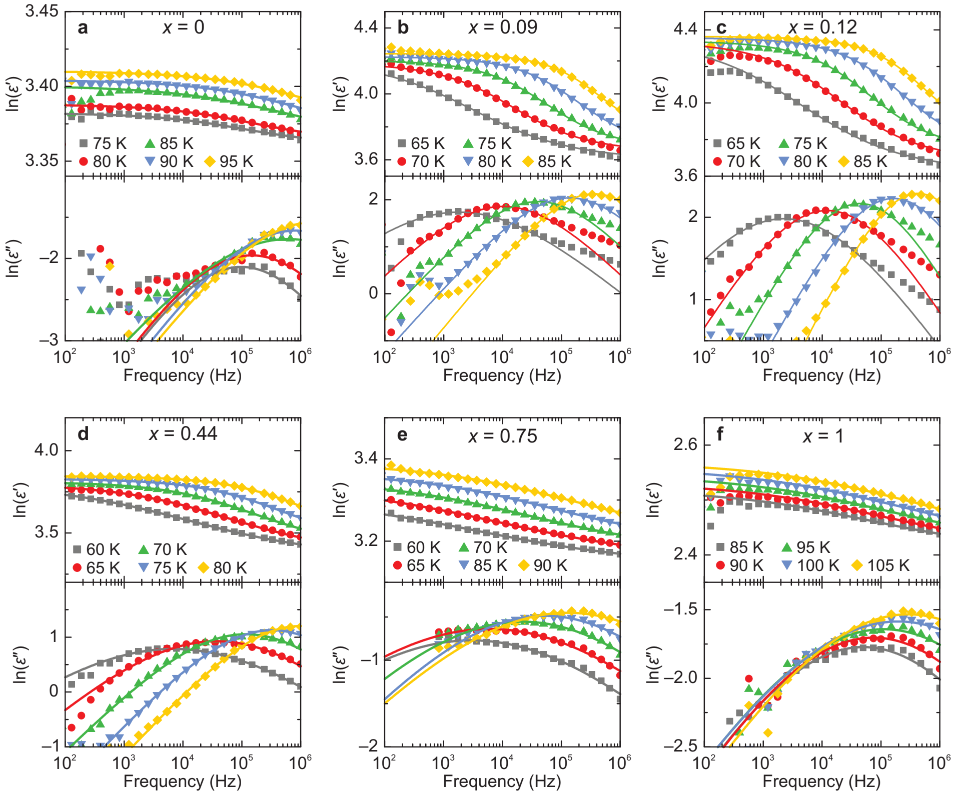

Figure S4: Frequency dependence of the complex dielectric permittivity of the τ_1 relaxation process of $MAPb_{1\text{-}x}Sn_xBr_3$ pellet samples presented at selected temperatures. The solid curves are the best fits to a single Cole-Cole relaxation process with the parameter α varying from 0.4 to 0.7.

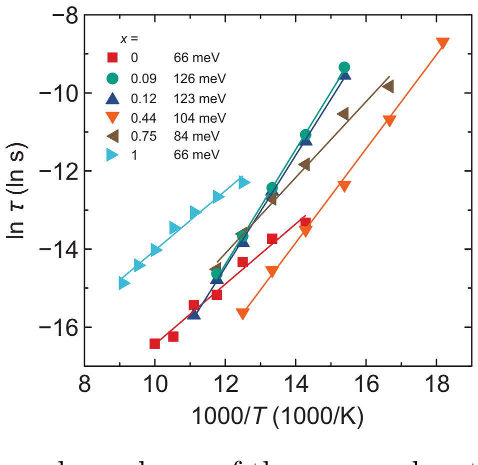

Figure S5: Inverse temperature dependence of the mean relaxation time of the τ_1 relaxation process observed in $MAPb_{1\text{-}x}Sn_xBr_3$ pellet samples measured by the dielectric spectroscopy. The solid lines indicate fits to the Arrhenius equation.

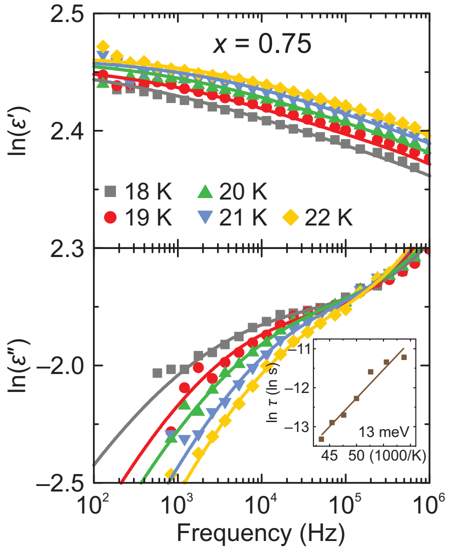

Figure S6: Frequency dependence of the complex dielectric permittivity of the τ_2 relaxation process of the $x = 0.75$ pellet sample. The solid curves are the best fits to two overlapping Cole-Cole relaxation processes. Inset shows the inverse temperature dependence of the obtained mean relaxation time. The solid line indicates the best fit to the Arrhenius equation.

References

(1) Schonhals, A.; Kremer, F. *Broadband Dielectric Spectroscopy*, 1st ed.; Springer-Verlag Berlin Heidelberg, 2003.
